# The Impact of Healthcare Pressures on the COVID-19 Hospitalisation Fatality Risk in England

**DOI:** 10.1007/s44197-024-00310-9

**Published:** 2024-10-08

**Authors:** Jonathon Mellor, Owen Jones, Thomas Ward

**Affiliations:** UK Health Security Agency, 10 South Colonnade, Poplar, London, UK

**Keywords:** Hospitalisation fatality risk, SARS-CoV-2, Hospital strain, Severity estimate

## Abstract

**Background:**

As the impact of the SARS-CoV-2 pandemic extends into 2023 and beyond, the treatment and outcomes of infected patients continues to evolve. Unlike earlier in the pandemic there are now further infectious disease pressures placed on hospitals, which influence patient care and triage decisions.

**Methods:**

The manuscript uses individual patient records linked with associated hospital management information of system pressure characteristics to attribute COVID-19 hospitalisation fatality risks (HFR) to patients and hospitals, using generalised additive mixed effects models.

**Results:**

Between 01 September 2022 and 09 October 2023, the COVID-19 hospitalisation fatality risk in England was estimated as 12.71% (95% confidence interval (CI) 12.53%, 12.88%). Staff absences had  an adjusted odds ratio of 1.038 (95% CI 1.017, 1.060) associated with the HFR when accounting for patient and hospital characteristics.

**Interpretation:**

This observational research presents evidence that a range of local hospital effects can have a meaningful impact on the risk of death from COVID-19 once hospitalised and should be accounted for when reporting estimates. We show that both the patient case mix and hospital pressures impact estimates of patient outcomes.

**Supplementary Information:**

The online version contains supplementary material available at 10.1007/s44197-024-00310-9.

## Introduction

Estimating the severity of a pathogen is crucial in effective public health protection, communications, and healthcare planning. Different choices in metrics tell us different information about pathogen severity and factors that impact patient outcomes. The case-fatality-risk (CFR) [1] gives the proportion of individuals identified with the disease who later die, widely reported during the SARS-CoV-2 pandemic [2, 3]. Cases are often detected following healthcare seeking behaviour or symptom presentation, which biases estimates toward more severe disease. The infection-fatality-risk avoids case ascertainment bias by randomly sampling the community population regardless of symptomatic status, with numerous examples during the pandemic [4, 5]; but these are costly and rare in practice. The hospitalisation-fatality-risk (HFR) gives a severity metric with lower ascertainment bias than the CFR, given high ascertainment in secondary care settings. However, severity estimates conditional on hospitalisation are not free from bias, as triage decisions and hospital strain play a role in patient outcomes.

Admission triage is dependent on many elements [6] including patient risk factors, such as age [7], comorbidities and the on-the-ground situation [8]. Hospitals perform mutual aid, though there is a limit to resources available, impacting triage decisions [9]. Triage impacts who is admitted, but in addition, hospitals undergo pressures and strain which impact patient care and outcomes, though the relationship is complex [10]. While high demand can be prepared for [11], increased hospital pressure is associated with higher mortality [12]. During particularly acute strain for a health system, such as a pandemic or challenging winter season, the quantity of sick patients increases, and staff to care for them decreases due to illness related absences. By exploring fatality risk based on a cohort of well ascertained admitted patients we can understand both the individual patient characteristics that influence fatality risk, but also the organisational strains and their role in outcomes.

Previous research has shown factors impacting the COVID-19 HFR early in the pandemic in England [13, 14], and elsewhere [15]. We explore the impact of case mixes and new hospital pressures on the COVID-19 HFR in England during later Omicron waves and an influenza season. We adjust for patient characteristics, hospital characteristics, and time-varying hospital strain – specifically due to COVID-19 bed occupancy, influenza bed occupancy, and staff absences.

## Methods

To estimate the COVID-19 HFR for different patient cohorts, hospitals, and to adjust for pressures, a range of data sources were linked. This retrospective cohort study includes patients who were admitted to acute hospitals in England between 01 September 2022 and 09 October 2023 with COVID-19.

### Data

#### Patient Level Characteristics

Admitted patient data are used to estimate the HFR, sourced from the Secondary-Use-Services (SUS) Emergency Case Data Set (ECDS) [16] and Admitted Patient Care [17] datasets from National Health Service (NHS) England. Using the Second Generation Surveillance System (SGSS) [18] from the UK Health Security Agency (UKHSA), COVID-19 positive tests are linked to the patient-level data from SUS. Hospitalisation data sets are truncated by 3 months to account for time to death and record upload. We define a COVID-19 admission as a patient admitted via emergency care, with a COVID-19 ICD-10 code and a positive linked test with specimen date between 14 days pre-admission and 5 days post-admission. This excludes probable hospital-acquired-infections.

From the SUS data, the patient’s age group, sex, and Charlson Comorbidity Index (CCI) [19] at the time of admission are captured. The age groups cover paediatrics, school age children, intervals of middle-aged and elderly adults. Vaccination records from National Immunisation Management System (NIMS) [20] give the vaccination dose number and Clinically Extremely Vulnerable status (CEV) [21]. The chosen outcome is, given hospitalisation with COVID-19, whether COVID-19 was the cause of death (mentioned in the registration) or death was within 28 days of specimen date, using ONS death registrations. The specimen to death date distributions are given in Supplementary Fig. 1.

As a retrospective observational study using routinely captured anonymised patient information and management information, patient and public involvement was not considered for the design of this study.

#### Trust Level Characteristics

A secondary care NHS Trust is a collection of hospitals in a local area with a shared management and resourcing structure, typically with 1 to 4 hospitals. In this study we include only acute secondary care Trusts with emergency wards, excluding specialist Trusts, defined by the Estates Returns Information Collection (ERIC) dataset [22], giving 119 Trusts in the cohort. Each Trust is unique, however, as a proxy for size we use the Trust’s catchment population as each Trust ranges in size and demographics [23]. In addition, we include the total clinical staff and total beds using NHS Workforce Statistics [24]. The distribution of catchment populations, clinical staff, and beds are given in Supplementary Fig. 2.

#### Pressure Characteristics

Some pressures on healthcare systems are consistent or have predictable patterns. Others—such as, infectious diseases, extreme weather, or natural disasters—exacerbate burden as patient load increases above anticipated demand [25]. Healthcare demand increases over the winter period in England, due to a mix of these pressures. Using the NHS England Urgent and Emergency Care (UEC) Situational Report [26] we identify pressures over time, within each Trust. The data: counts of staff absences, beds occupied with influenza patients, and beds occupied with COVID-19 patients are dependent on the size of the different Trusts. Therefore, we model with predictors per Trust: COVID-19 patient occupied beds / total beds, influenza patient occupied beds / total beds, absent staff / total staff, where the total is by Trust.

The decision to admit, and quality of care can influence fatality risk throughout the patient’s stay, therefore, multiple pressure statistics were explored in development of these models. The pressures metric selected was the value on the day of the patient’s admission. Further data processing is outlined in Supplementary Sect. 1.

#### Model Effect Structure

**Fig. 1:**
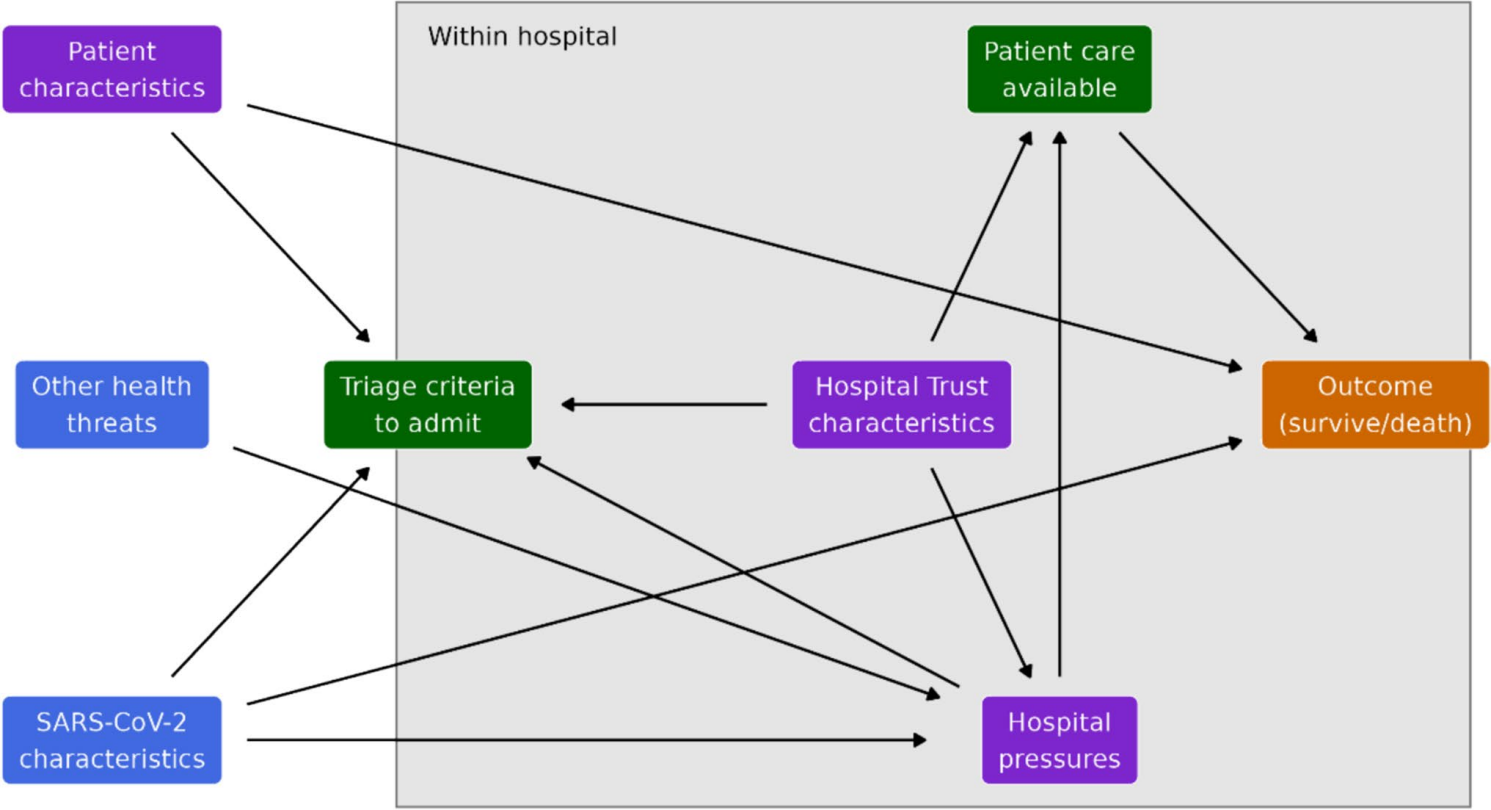
Shows the Directed Acyclic Graph (DAG) of the hospitalisation fatality risk in this study. Partially measured concepts are given in purple (Patient characteristics, Hospital Trust characteristics, Hospital pressures), the outcome in orange (survive or death), unmeasured effects in green (Triage criteria to admit, Patient care available) and time varying community effects outside of the hospital (SARS-CoV-2 characteristics, other health threats) in blue. The arrows indicate the direction of relationship expected

There are numerous effects in the hospital system and disease dynamics influencing patient fatality risk. Patient, hospital, and pressure characteristics will impact patient deterioration directly and indirectly. Our anticipated causal structure is outlined as a Directed Acyclical Graph (DAG) (Fig. 1).

We expect that the outcome of death is directly impacted by three main factors, and moderated by other effects. Frail or vulnerable patients are more likely to die, therefore the patient’s characteristics directly impact fatality risk even conditioned on hospitalisation. The patient care (such as equipment, staff time or expertise available) changes the patient's COVID-19 fatality risk, with less care increasing risk. The characteristics of SARS-CoV-2 itself should directly alter the fatality risk as different variants cause different immune responses.

The decision to admit a patient introduces a selection bias that confounds COVID-19 HFR estimates. When higher proportions of frail patients are admitted, the HFR inflates. Conversely, more young or fewer comorbidities patients may reduce the observed HFR. In high pressure periods we expect admission criteria to select more severe patients, raising the HFR. We expect the triage decision is directly influenced by patient characteristics presented to the clinicians, with higher risk patients more likely to be admitted. To adjust for this case mix confounding effect we include individual patient characteristics that influence the admission decision. Futhermore, if a new SARS-CoV-2 variant grows in prevalence, this may change a clinicians admittion decision for COVID-19 patients. Additionally, the characteristics of a hospital (such as baseline staffing, specialisation and size) will impact admission triage.

We cannot directly measure patient care available—it is highly complex, however, we can measure some pressures which may impact patient care, namely beds occupied by patients with some infectious diseases and staff absences. We expect these pressures to increase risk by reducing the patient care available, though these pressures also impact the descision to admit. We expect there to be residual confounding due the non-measured hospital strain impacting patient care available. Furthermore, we cannot adjust for changing SARS-CoV-2 characteristics, but we allow an effect to vary smoothly over time, as with other health threats that cause hospital pressures.

We assume that external health threats impact the COVID-19 HFR through hospital capacity by introducing a new pressure (such as emergency wards being full, reduced availability of staff). These effects cannot directly be adjusted for as sharp system shocks and are assumed as residual confounding. SARS-CoV-2 severity and population immunity (due to reinfection) are expected to change over time and impact the HFR. The individual infection history and population severity estimates are not available, therefore we include a slow moving temporal effect adjusting for these charactertics. This effect may also adjust partially for other slow moving confounders, such as non-modelled pressures. Each hospital Trust is different, with different characteristics, local practices and specialisations, which we expect to indirectly impact the HFR via the triage decisions, pressures created and care available.

#### Model Form

The COVID-19 HFR can be characterised as a probability of an admitted patient dying or surviving following a hospital admission with SARS-CoV-2. At an individual level, the outcome ${Y}_{i}$ is either death or survival of those admitted, a binary response with some predictors ${x}_{j,i}$ given by $j=\text{1,2},\dots , m$. With a Bernoulli distribution on the probability of an outcome ${\text{p}}_{i}$, the HFR, we obtain

$${Y}_{i}| {x}_{1,i}\dots {x}_{m,i} \sim {\text{Bernoulli}}({\text{p}}_{i})$$

using the logistic function with ${\text{p}}_{i}$ and the linear predictors with coefficients $\upbeta_j$

$$\text{logit}\left({\text{p}}_{i}\right)={\upbeta }_{0}+{{\upbeta }_{1}{ x}_{1}+\dots +\upbeta }_{m} {x}_{m}$$

gives our regression equation. To estimate the coefficients, we employ a generalised additive mixed effect model framework allowing the addition of random effects for the predictors ${x}_{j}$ denoted by ${f}_{RE}({x}_{j})$ and smooth function denoted ${f}_{s}({x}_{j})$. The models are hierarchical, covering both NHS Trust level effects, and individual patient effects.

The models are fit using the R *mgcv* package [27] and restricted maximum likelihood (REML). We extracted the model’s predictions using the *marginaleffects* R package [28], and odd-ratios from the *modelsummary* package [29]. Several models were developed demonstrating the difference in fit between structures. To compare model goodness of fit we use the Akaike Information Criteria (AIC) and Bayesian Information Criteria (BIC).

#### Specific Models

We start with a simple model, assuming that the HFR is determined by the patient’s characteristics: age group, sex and comorbidities (CCI), vaccine dose and COVID Extremely Vulnerable (CEV) and a time-varying spline ${f}_{s}(t)$, where $t$ is the admission date. A day of week $(\text{dow})$ on admission date random effect ${f}_{RE}(\text{dow})$ is included. A random effect per patient is given to adjust for repeated admission ${f}_{RE}\left(\text{patient}_i\right)$. This “individual” model is given by:

$${Y}_{i}\sim \text{Bernoulli}({\text{p}}_{i})$$

$${\text{logit}}\left({\text{p}}_{\text{i}}\right) = {\upbeta }_{0} + {\upbeta }_{1} {\text{age group}}_{i} + {\upbeta }_{2} {\text{sex}}_{i}+{f}_{\text{RE}}\left(\text{dow}\right)+{f}_{RE}\left(\text{patient}_i\right)+{f}_{s}(t)$$

$${\upbeta }_{3} {\text{CCI}}_{i} + {\upbeta }_{4} {\text{dose}}_{i} + {\upbeta }_{5} {\text{CEV}}_{i}$$

Building upon the “individual” model comes the “trust” hierarchical model, with a random intercept for each Trust ${f}_{RE}({\text{trust}}_{i})$, allowing for variation in location, the size of the Trust catchment $\text{population}$, and ratio of total clinical staff to total beds $\frac{{\text{staff}}_{\text{i}}}{{\text{beds}}_{\text{i}}}$. The “trust” model’s equation becomes:

$${\text{logit}}\left({\text{p}}_{i}\right)={\upbeta }_{0} + {\upbeta }_{1} {\text{age group}}_{i} + {\upbeta }_{2} {\text{sex}}_{i}+{f}_{\text{RE}}(\text{dow})+{f}_{RE}\left(\text{patient}_i\right)+{f}_{s}(t)$$

$${\upbeta }_{3} {\text{CCI}}_{i} + {\upbeta }_{4} {\text{dose}}_{i} + {\upbeta }_{5} {\text{CEV}}_{i}$$

$$+{\upbeta }_{6}\frac{{\text{population}}_{i}}{\text{100,000}}+{\upbeta }_{7}\frac{{\text{staff}}_{i}}{{\text{beds}}_{i}}{ + f}_{RE}({\text{trust}}_{i}).$$

The “pressures” model builds upon the “trust” model, by adding an ${x}_{j}$ term for three time varying pressures, given by:

$$\text{logit}\left({\text{p}}_{i}\right)={\upbeta }_{0} + {\upbeta }_{1} {\text{age group}}_{i} + {\upbeta }_{2} {\text{sex}}_{i}+{f}_{\text{RE}}(\text{dow})+{f}_{RE}\left(\text{patient}_i\right)+{f}_{s}(t)$$

$${\upbeta }_{3} {\text{CCI}}_{i} + {\upbeta }_{4} {\text{dose}}_{i} + {\upbeta }_{5} {\text{CEV}}_{i}$$

$$+{\upbeta }_{6}\frac{{\text{population}}_{i}}{\text{100,000}}+{\upbeta }_{7}\frac{{\text{staff}}_{i}}{{\text{beds}}_{i}}{ + f}_{RE}\left({\text{trust}}_{i}\right)$$

$$+{\upbeta }_{8} {\text{flu}}_{i}+{\upbeta }_{9} {\text{covid}}_{i}+{\upbeta }_{10} \text{staff} {\text{ absence}}_{i}.$$

#### Conditional Risk

We will initially express the COVID-19 conditional on the whole cohort, the average result given those admitted in the study period. In addition, we can express the HFR as a marginal effect on different characteristics (such as age), by averaging over the population in question. However, the population cohort of those admitted with COVID-19 is not static over time – it depends on triage decisions. We define a conditional HFR as an estimate based on a specific cohort at a point in time, namely a single month of admissions. We take an “observed” HFR as a naïve model that produces an average HFR over a month period, a categorical variable ${\text{t}}_{\text{month}.\text{year}}$ gives,

$$\text{logit}\left({\text{p}}_{i}\right)={\upbeta }_{11} {t}_{\text{month}.\text{year}.}$$

A well-adjusted model should produce a similar conditional estimate HFR to the observed HFR each month.

## Results

### Descriptive Analysis

There were 132,057 admissions meeting the selection criteria, of 76,176 unique patients, with 16,779 corresponding fatalities from patients attending 119 Trusts. Patients were more likely to be 65 + , Male, with a CCI greater than 0, with 4 + vaccine doses and not CEV, given in Table 1. The time varying national proportion of each age group and CCI are shown in Supplementary Fig. 4, showing evidence of changing patient mix over the study period, particularly at times of low overall COVID-19 admissions.

**Table 1:** Counts and proportions of the study cohort. A record in this analysis is an admission episode, where there can be multiple admission episodes per person over the study period

Characteristic	Strata	Count of admission episodes	Proportion of all admission episodes (%)	Count of unique patients	Proportion of unique patients per admission episode (%)
	Admissions	132,057	–	76,176	57.7
	Fatalities	16,779	12.7	–	–
Age group	00–04	4,904	3.71	4,396	89.6
	05–17	1,177	0.892	988	83.9
	18–34	4,627	3.51	3,299	71.3
	35–54	10,064	7.64	6,227	61.9
	55–64	11,876	9.00	6,945	58.5
	65–74	22,518	17.0	12,576	55.8
	75–84	40,291	30.5	22,006	54.6
	85 +	36,600	27.7	19,755	54.0
Sex	Female	63,162	47.8	37,116	58.8
	Male	68,895	52.2	39,060	56.7
CCI	0	25,870	19.6	18,069	69.8
	1–2	70,540	53.4	39,874	56.5
	3–4	30,937	23.4	16,245	52.5
	> = 5	4,710	3.57	2,376	50.4
Dose number	0	14,739	11.2	10,324	70.0
	1–3	33,104	25.1	19,169	57.9
	4 +	84,214	63.7	46,694	55.4
Clinically extremely vulnerable	No	92,225	30.2	54,279	58.9
	Yes	39,832	69.8	21,897	55.0

The proportion of all admission episodes gives how many times that individuals in that strata appear in all episodes. The proportion of unique patients per admission gives the number of unique patients in a strata divided by the number of episodes within that strata. There are 119 unique Trusts within the study

Before modelling, the trends, and scales of variables of interest should be understood over the study period. The counts of total admissions and deaths are given in Supplementary Fig. 3, with a cut-off defined for the admission date, as deaths trail admissions. There are three complete admissions waves, with a partially complete wave at the end of the time series.

**Fig. 2:**
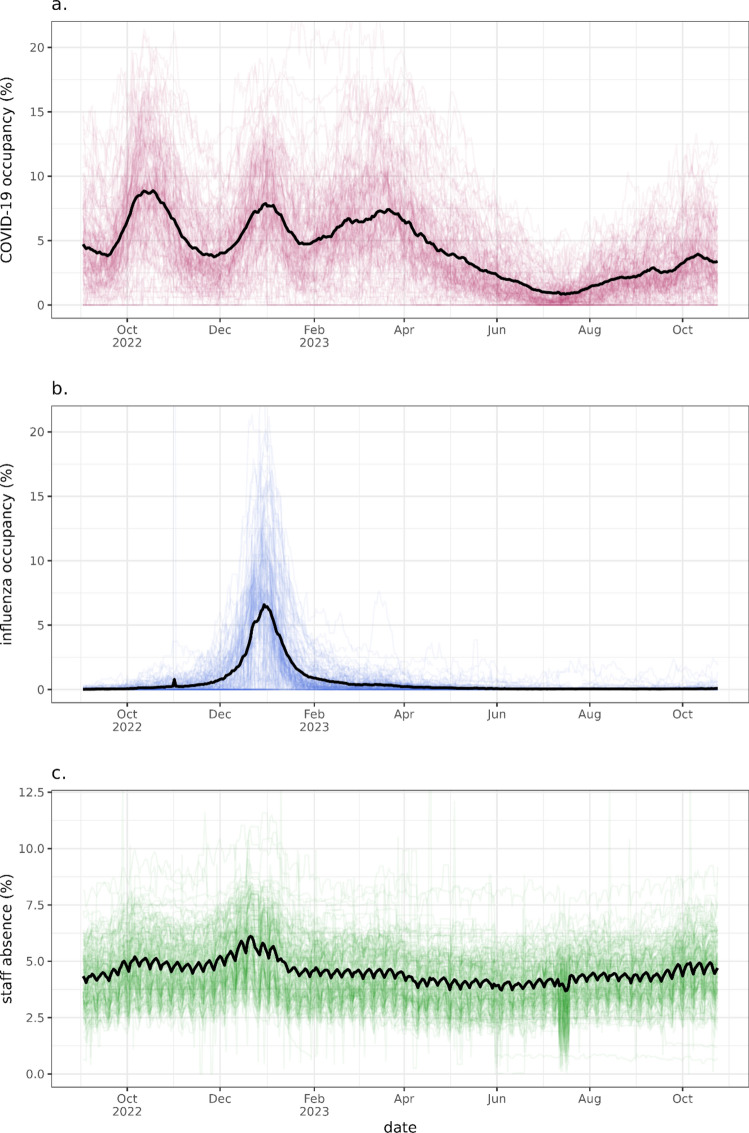
The time-varying value of the three pressures: COVID-19 occupancy, influenza occupancy and staff absence. The black line indicates the mean value across Trusts by day. Each line presents one Trust’s value over time, highlighting general trend the variation in measured values

The pressures on a hospital investigated in this work (beds occupied by test confirmed COVID-19 and influenza patients, as well as staff absences) vary across Trusts and time. The 2022/2023 influenza season was an intense year with a high but short-lived peak in occupancy. COVID-19 progressed in successive waves across the period without a clear periodicity. The variation by Trust will allow us to explore the effects via regression, shown nationally (Fig. 2) and regionally (Supplementary Fig. 5). These pressures data are rapidly collected management information, and measurement quality therefore varies.

### Model Estimates

The odds ratio of effects in each model are given in Table 2. The corresponding *p-values* are given in Supplementary Table 2 as well as further goodness-of-fit measures in Supplementary Table 3.

**Table 2:** Odds ratio from each model showing the relative risk of each effect

	Odds ratio [95% confidence interval]		
	Individual	Trust	Pressures
Age group: 00–04	0.008 [0.003, 0.021]	0.008 [0.003, 0.021]	0.008 [0.003, 0.021]
Age group: 05–17	0.070 [0.036, 0.135]	0.072 [0.037, 0.139]	0.072 [0.037, 0.139]
Age group: 18–34	0.104 [0.079, 0.137]	0.106 [0.081, 0.140]	0.106 [0.081, 0.140]
Age group: 35–54	0.372 [0.337, 0.411]	0.377 [0.342, 0.417]	0.377 [0.342, 0.417]
Age group: 55–64	0.630 [0.586, 0.677]	0.638 [0.593, 0.686]	0.638 [0.593, 0.686]
Age group: 65–74	0.783 [0.744, 0.824]	0.787 [0.747, 0.828]	0.787 [0.747, 0.828]
Age group: 85 +	1.469 [1.413, 1.528]	1.479 [1.423, 1.538]	1.479 [1.423, 1.538]
Sex: Female	0.805 [0.778, 0.832]	0.809 [0.782, 0.836]	0.808 [0.781, 0.836]
CCI: > = 5	1.784 [1.659, 1.918]	1.823 [1.693, 1.962]	1.823 [1.693, 1.962]
CCI: 0	0.524 [0.491, 0.559]	0.522 [0.489, 0.558]	0.522 [0.489, 0.558]
CCI: 3-4	1.397 [1.346, 1.450]	1.410 [1.358, 1.463]	1.410 [1.359, 1.464]
Dose number: 0	1.391 [1.295, 1.493]	1.456 [1.355, 1.564]	1.454 [1.354, 1.563]
Dose number: 1–3	1.167 [1.119, 1.218]	1.186 [1.136, 1.239]	1.187 [1.137, 1.240]
Clinically extremely vulnerable	1.155 [1.114, 1.197]	1.169 [1.128, 1.212]	1.169 [1.128, 1.212]
Catchment population/100,000	–NA–	0.979 [0.959, 0.998]	0.980 [0.961, 1.000]
Total clinical staff/Total beds	–NA–	0.956 [0.922, 0.992]	0.963 [0.929, 0.999]
COVID-19 occupancy (%)	–NA–	–NA–	1.000 [0.994, 1.006]
Staff absence (%)	–NA–	–NA–	1.038 [1.017, 1.060]
Influenza occupancy (%)	–NA–	–NA–	1.008 [1.000, 1.015]
Average HFR	12.71% [12.53%, 12.88%]	12.71% [12.53%, 12.88%]	12.71% [12.53%, 12.88%]
Observations	132,057	132,057	132,057
AIC	94,140.7	93,577.8	93,559.8
BIC	94,330.9	94,736.8	94,744.0

The reference classes were chosen as the modal class for each predictor, taken as Age group: 75–84, Sex: Male, CCI: 1–2, Dose number: 4 + . The AIC and BIC goodness-of-fit measures are given for each model

Using the “pressures” model, we estimate an overall HFR of 12.71% (95 CI 12.53%, 12.88%). On average a patient admitted with COVID-19 has an approximately 1-in-8 chance of dying. This is not a case or infection-fatality risk, it is conditioned on an individual being admitted, and therefore already presenting severely with the disease. Patient characteristics, pressures, hospital characteristics, and timing do influence this HFR in different ways, which we explore in greater detail.

**Fig. 3:**
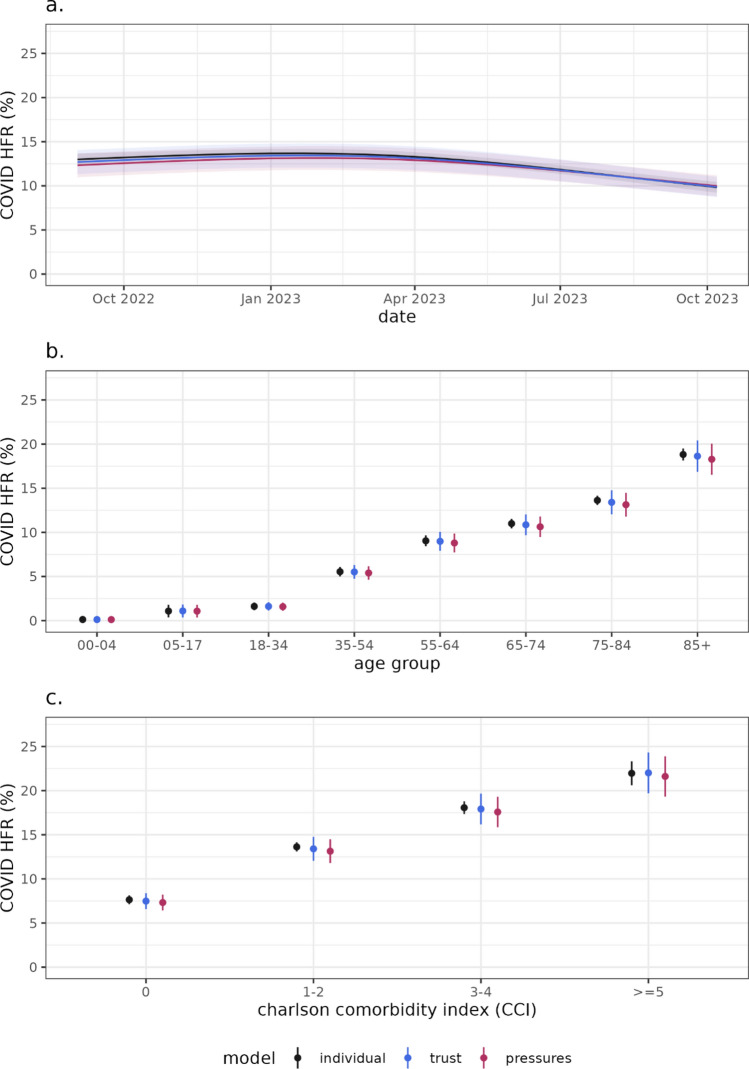
The marginal effect of time (**a**), age group (**b**) and Charlson comorbidity index (**c**) on the HFR for the estimated model. As the covariate is varied (on the x-axis) we can see how the response (y-axis) varies, while keeping all other covariates in the model constant at their average value

We first compare key variables included in all models, the time varying effect, age group, and comorbidity index (Fig. 3). The time varying spline marginal effect (Fig. 3a) indicates a higher HFR over the winter and spring, declining as the time series goes on to lower values over the summer months. There is a clear age gradient in the HFR, with expected higher fatality risks for older patients (Fig. 3b). More comorbidities (Fig. 3c) and being CEV are associated with a higher marginal HFR (Table 2). Lower vaccination is associated with higher HFR across models. For the “trust” and “pressures” model, the catchment population and the clinical staff to bed ratio have some evidence of an impact on the HFR (Supplementary Table 2) and high variation in the Trust effect (Supplementary Fig. 6).

**Fig. 4:**
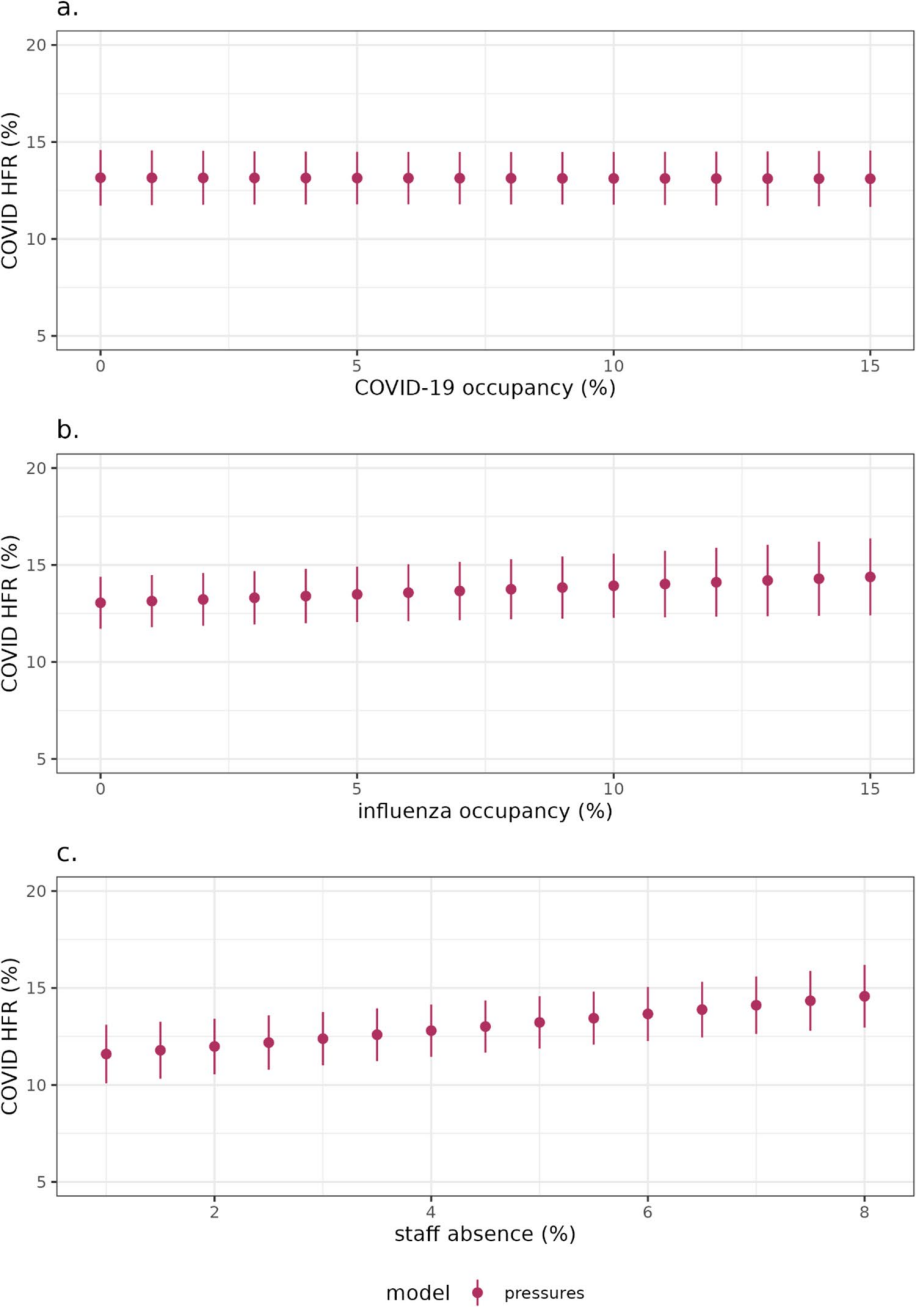
The marginal effect of the maximum pressure during a COVID patient’s stay of COVID bed occupancy (**a**), influenza bed occupancy (**b**) and staff absences (**c**) on the HFR in the estimated model. As the covariate is varied (on the x-axis) we can see how the response (y-axis) varies, while keeping all other covariates constant

We explore the relationship between the HFR and different hospital pressures for the “pressures” model (Fig. 4). There is no clear relationship between the COVID-19 bed occupancy and the HFR (Fig. 4a). Influenza bed occupancy is low throughout the year, spiking in winter, with a small increase in marginal HFR related to the influenza occupancy pressure (Fig. 4b). There is a clearer gradient to the effect of staff absence on the HFR—as staff absence percentages increases so does the HFR, after adjustment for other variables (Fig. 4c)*.*

**Fig. 5:**
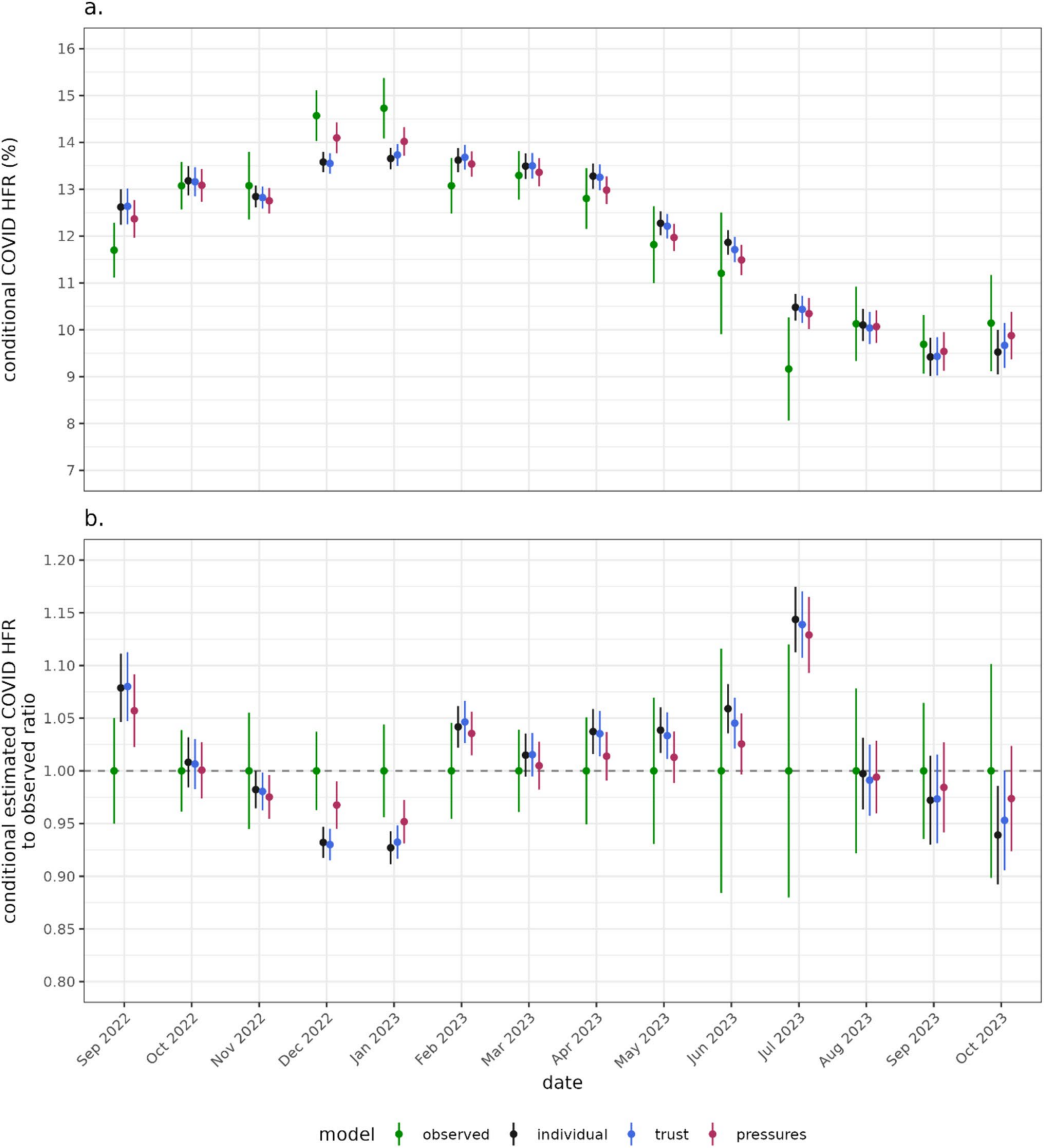
**a** The average estimated HFR and observed HFR for patients that were admitted each month. **b** The ratio between the estimated HFR each month and the observed HFR that month. The observed HFR gives an estimate conditional on the case mix as it varied over time. Estimates closer to the observed HFR capture the population level dynamic better

Another lens to explore the model performance and utility is to see whether the models can reproduce the observed conditional HFR at different points in the study period. The average HFR is given for patients in each month (Fig. 5), comparing between the observed and estimated model HFR. The naïve “observed” HFR estimate varies substantially over the time series, higher over winter than summer, corresponding to the admission counts (Supplementary Fig. 3). The “individual” model adjusts for patient level characteristics, a slow changing temporal effect and the day of week. This “individual” model adjusts for the case mix, but not hospital pressures. The “pressures” model more accurately predicts the observed HFR in 12 of 14 months, particularly over winter.

## Discussion

The results show numerous effects influence the COVID-19 HFR. By accounting for patient characteristics, temporal effects, hospital qualities and pressures the HFR was estimated with appropriate adjustment, giving an overall HFR of 12.71% (95% CI 12.53%, 12.88%). Key quantities impacting the HFR include patient age, comorbidities, vaccine dose number, sex, and vulnerability status. By adjusting for these, we explore the impact of hospital pressures and understand their effects on individual outcomes.

The modelled estimates agree with earlier HFR literature for COVID-19. Older patients with more comorbidities and fewer vaccinations have higher risk of dying, even conditional on an admission triage [13, 30]. Measured patient characteristics allow us to statistically adjust for the patients being admitted, and therefore the cohort and case mix experienced by the hospitals, which varies over time. Because of this adjustment we can explore how other phenomena impact the HFR, namely hospital level effects.

Using the AIC the “pressures” model most parsimoniously fits the data compared to the “trust” and “individual” models, with scoring shown in Supplementary Table 3. However, the BIC for the “individual” model is preferred. This an artifact of the definition of each criteria, and the extent they penalise parameter quantity and sample size, with the BIC penalising proportional to sample size. However, the “pressures” model better captures the time varying conditional HFR, particularly during the winter when influenza and staffing levels introduced high concurrent pressures on healthcare services (Fig. 5). This result indicates hospital pressures are particularly important to account for when hospital systems are under heavy strain, as the changing HFR cannot be explained by case mixes alone.

We anticipate the hospital pressures impact both triage decisions (forcing a selection bias) and the care given to individuals, however, by adjusting for patient characteristics we mediate the effect of triage decisions on the HFR, giving a more accurate estimate of the influence of the pressures directly on the risk. To account for time varying characteristics of SARS-CoV-2, changes to population immunity and other health threats we included a temporal smooth effect. Hospital pressures may have changed on a similar characteristic time scale to this slow-moving effect, which could moderate their effect size. The inclusion of a Trust random effect allows us to account for some baseline HFR per hospital and partially adjust for baseline pressure levels varying by Trust (such as persistent high staff absence), supporting the claim that the pressures influence the HFR directly.

The pressure shown with highest impact on the HFR is staff absences on the individual’s day of admission. Staff absences reduce care available to patients and/or cause redeployment of staff to areas they are less experienced in to account for shortfalls. Staff absences at a Trust level will be impacted by a multitude of reasons including illness, both acute (including COVID-19 itself) and chronic, and other stochastic events.

The COVID-19 HFR is an important severity metric to understand for hospital services as the severely ill require more care. In this study we include deaths beyond the hospital system, using linked death certificates and timing of deaths to ensure probable and causative COVID-19 deaths are included, as patients may be discharged for palliative care due to their infection. Within hospital mortality alone would underestimate risk to patients and alter adjusted odds ratios as some patients are more likely to be discharged for palliative than others.

The desire to untangle a wide range of effects led us to choose a hierarchical generalised additive model logistic regression. A range of alternative analytical approaches were possible, though some approaches were limited by software available. Instead of logistic regression with a binary outcome, a survival approach such as a proportional hazards model would have been appropriate to adjust for the right censoring in the data and given a time-to-death estimate. This right censoring was not accounted for in our approach due to completeness of the data given the time elapsed. A smooth-function of time was chosen to model slow moving temporal changes in risk over the study period – instead fixed or random effects could be employed, such as a monthly indicator, to account for temporal variation, though this would not guarantee a smooth, autocorrelated effect over time. Further non-linearities could be added, such as an age-effect on the HFR, but non-linear effects are often challenging to interpret relative to adjusted-odds ratios of categorical variables.

While this work did include pressures not yet explored in the literature on COVID-19 HFRs, more could be explored, such as ambulance handover times, admission waiting times, all-cause bed occupancy and critical care pressures. However, healthcare systems and capacity are multi-faceted, especially under strain [31], we focused on obvious candidate pressures. This study includes admissions over the winter period in 2022/2023 up to autumn 2023. A longer time series would give more conclusive results, variation in SARS-CoV-2, and a second influenza season. Different COVID-19 testing policies and measurement of healthcare pressures pose challenges, limiting generalisability over long periods.

The observational unit of this study for hospital characteristics and pressures was the NHS Trust, which often contain multiple hospitals. More granular, local data on the hospital and ward may give a clearer picture on triage decisions and care procedures. In addition, further individual patient data such as vital signs would help more explicitly adjust for triage decisions. Understanding the effect of pressures at different points of a patient’s pathway would be interesting for future work.

SARS-CoV-2, immunity and other pressures are constantly evolving, with the healthcare system adapting to them, however, the methods shown in this work can be applied when newer data is available and different threats arise. This work accounted for the hierarchical structure of a health service with local pressures, and individual characteristics which could be more widely used in this type of risk modelling, as could non-linear terms be more widely used to partially adjust for unmeasured confounding that changes over time.

## Conclusion

In this work we estimate the HFR for COVID-19 patients in England, which includes concurrent infectious disease pressures period in 2022–2023. By adjusting for key patient characteristics, we show evidence of the effect of hospital pressures on the HFR estimate, implying higher system strain raises the HFR even when accounting for changing patient mix, particularly due to staff absences.

## Supplementary Information

Below is the link to the electronic supplementary material.

Supplementary file1 (DOCX 1978 KB)

## Data Availability

UKHSA operates a robust governance process for applying to access protected data that considers: the benefits and risks of how the data will be used, with policy, regulatory and ethical obligations, data minimisation, how the confidentiality, integrity, and availability will be maintained, retention, archival, and disposal requirements, best practice for protecting data, including the application of ‘privacy by design and by default’, emerging privacy conserving technologies and contractual controls ccess to protected data is always strictly controlled using legally binding data sharing contracts. KHSA welcomes data applications from organisations looking to use protected data for public health purposes. To request an application pack or discuss a request for UKHSA data you would like to submit, contact DataAccess@ukhsa.gov.uk.
