# Supplementary material for: The Impact of Healthcare Pressures on the COVID-19 Hospitalisation Fatality Risk in England
Source: J Epidemiol Glob Health. 2024 Oct 8;14(4):1579–90. doi: 10.1007/s44197-024-00310-9 (PMC11652468; doi:10.1007/s44197-024-00310-9)
Supplement: Supplementary file 1 — Supplementary file1 (DOCX 1978 KB) [file 44197_2024_310_MOESM1_ESM.docx]

**Supplementary information**

**
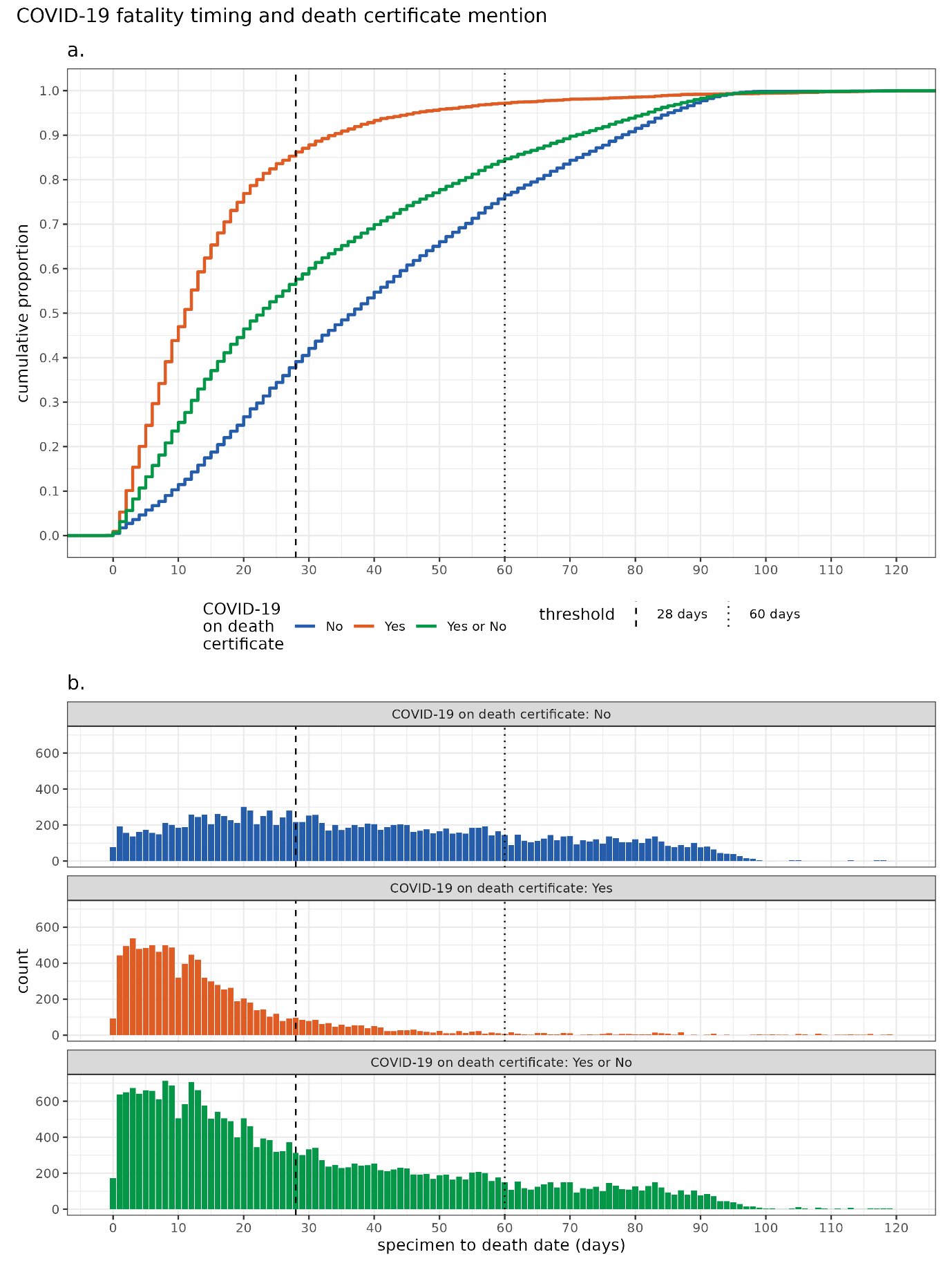
**

Supplementary Figure 1. The cumulative proportion of time to death from specimen date (a.) and total count by time to death (b.) of those admitted. Standard surveillance definition thresholds in England at 28 and 60 days from positive specimen date are given. The time delay is more left skewed for those deaths where COVID-19 appeared on the death certificate. Mentions of COVID-19 on the death certificate give causative deaths, however, they under count probable deaths due to COVID-19, motivating the additional use of time to event thresholds.


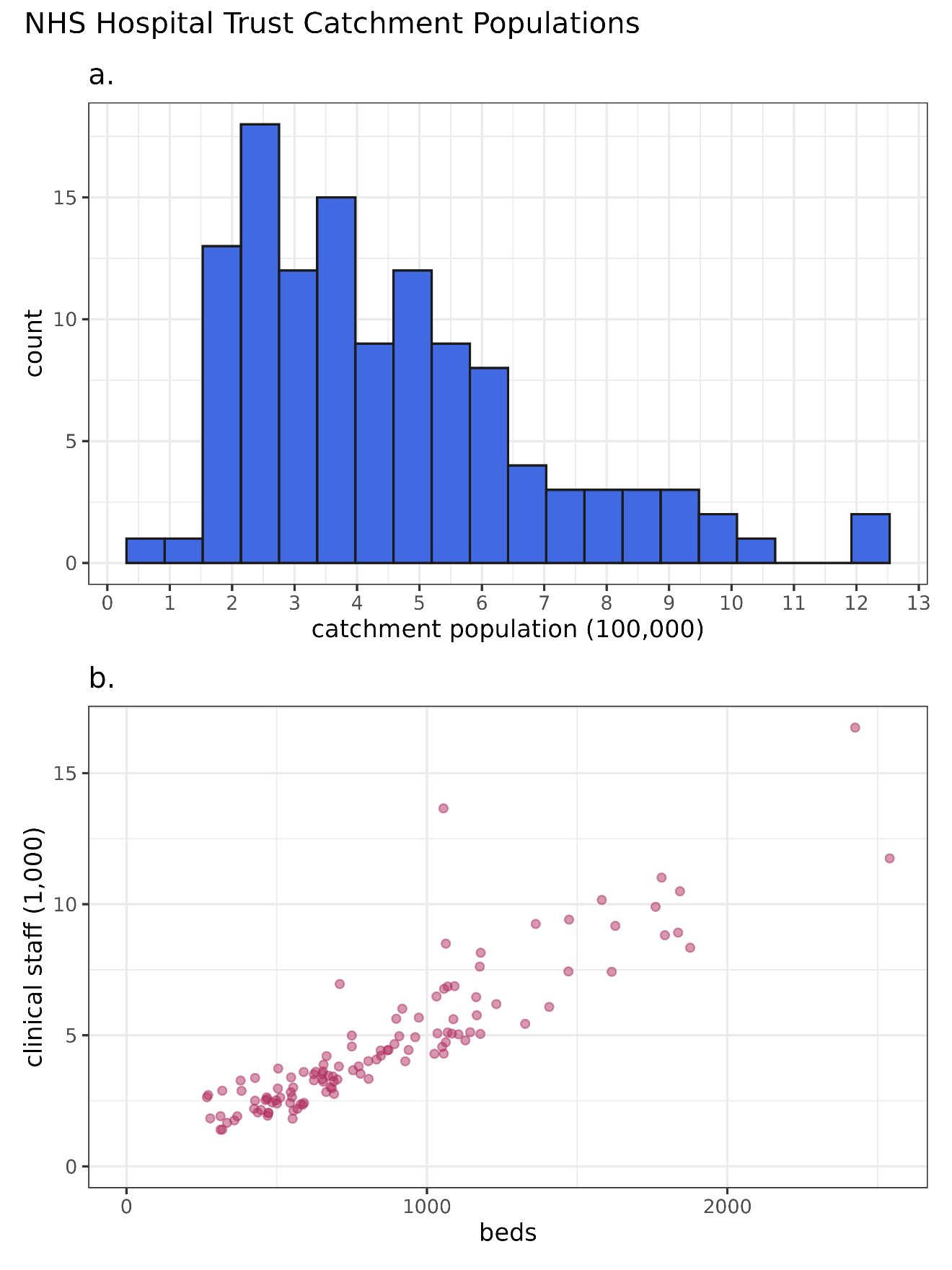


Supplementary Figure 2. The characteristics of the 119 acute Trusts reporting data for the study. The distribution of the Trust’s population catchment sizes (a.) and staff to bed ratios (b.) indicate the relative sizes of different Trusts across metrics.

**Supplementary Section 1 – Data Processing**

Records where any of the following key variables were missing, were excluded: linkage identifier, NHS Trust Code, age group, admission/discharge date, however very few of these entries were missing. Breakdowns of missing data are provided in Supplementary Table 1, the most missing values were found in the pressures variables. Implausible bed occupancy reports, such as a pressure greater than 100% or negative values, treated as missing. The pressures variables were imputed using the average normalised value per NHS region per day to capture a plausible trend where records were missing, the regional trends are given in Supplementary Figure 4.

| **Level** | **Variable** | **Missingness (%)** |
| --- | --- | --- |
| Individual | Trust code | 0 |
|  | Age group | 0 |
|  | CCI | 0 |
|  | Vaccine Dose | 0 |
|  | CEV | 0 |
|  | Admission date | 0 |
|  | Fatality | 0 |
| Trust | Total Clinical Staff | 0 |
|  | Total Beds | 0 |
|  | Population | 0 |
| Pressures | COVID bed occupancy | 0 |
|  | Influenza bed occupancy | 10.47 |
|  | Staff absence | 0 |

Supplementary Table 1. The missingness of each variable. As the final modelling data set is a combination of multiple data sets requiring joining on keys which may be missing, missing data are reported for those records with trust codes within the APC data set. The COVID bed occupancy and Staff absence data are from one UEC data stream, and the influenza bed occupancy from a second, causing their structural differences in missingness. Beds and total staff were considered missing if equal to zero or directly missing, pressures were considered missing if unavailable or greater than their denominator (beds and total staff).


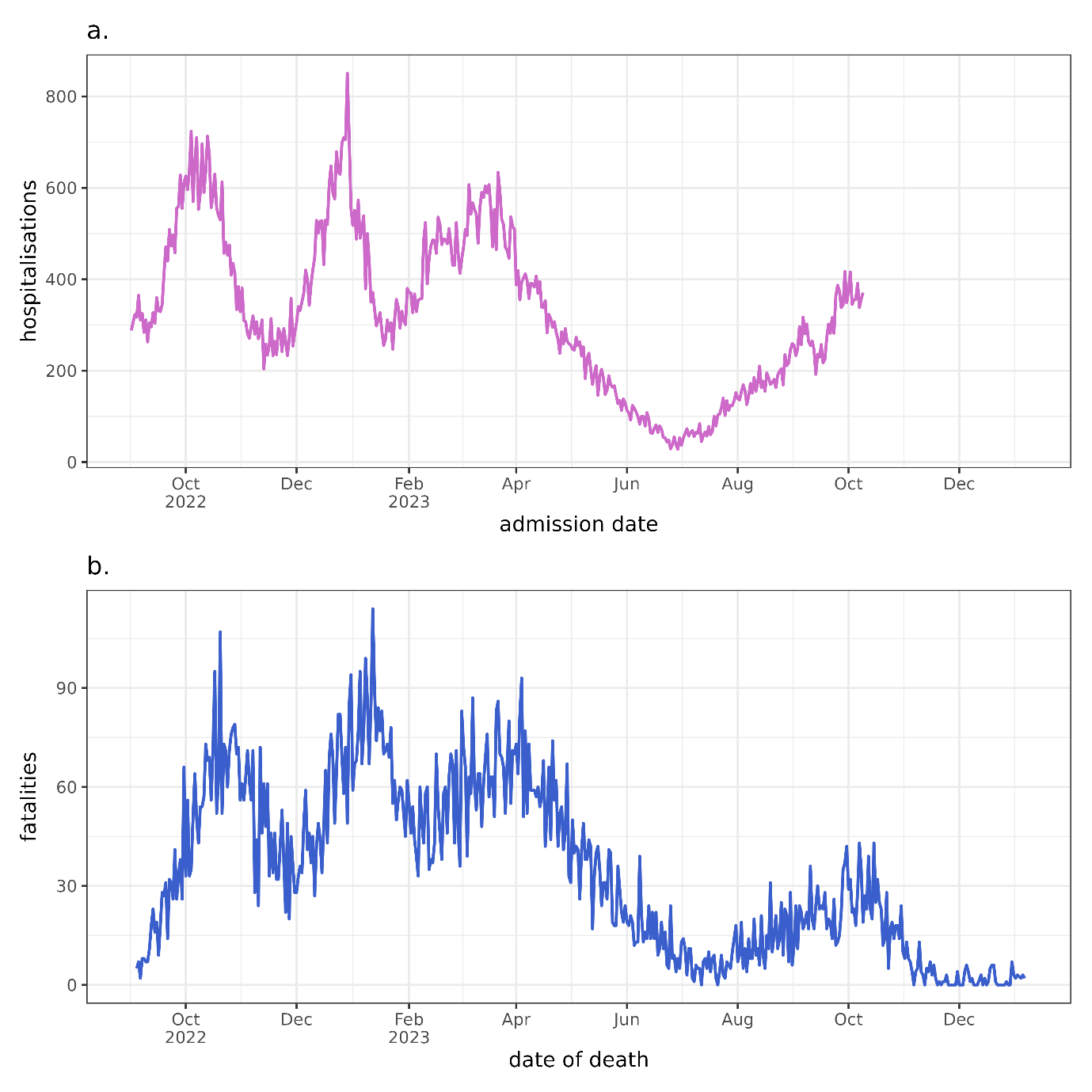


Supplementary Figure 3. The counts of hospitalisations (a.) and fatalities (b.) associated with patients in the study window 01 September 2022 to 09 October 2023 in England. There are many more admissions per day, as deaths are a subset of admitted patients. The admissions follow a trend that varies over epidemic wave and is impacted by day-of-week effect. The peaks in the deaths follow the admissions waves in time.

|  | **p-value** | | |
| --- | --- | --- | --- |
|  | **individual** | **trust** | **pressures** |
| Age group: 00-04 | <0.001 *** | <0.001 *** | <0.001 *** |
| Age group: 05-17 | <0.001 *** | <0.001 *** | <0.001 *** |
| Age group: 18-34 | <0.001 *** | <0.001 *** | <0.001 *** |
| Age group: 35-54 | <0.001 *** | <0.001 *** | <0.001 *** |
| Age group: 55-64 | <0.001 *** | <0.001 *** | <0.001 *** |
| Age group: 65-74 | <0.001 *** | <0.001 *** | <0.001 *** |
| Age group: 85+ | <0.001 *** | <0.001 *** | <0.001 *** |
| Sex: Female | <0.001 *** | <0.001 *** | <0.001 *** |
| CCI: >=5 | <0.001 *** | <0.001 *** | <0.001 *** |
| CCI: 3-4 | <0.001 *** | <0.001 *** | <0.001 *** |
| CCI: 0 | <0.001 *** | <0.001 *** | <0.001 *** |
| Dose number: 0 | <0.001 *** | <0.001 *** | <0.001 *** |
| Dose number: 1-3 | <0.001 *** | <0.001 *** | <0.001 *** |
| Clinically Extremely Vulnerable | <0.001 *** | <0.001 *** | <0.001 *** |
| Catchment population/100,000 | --NA-- | 0.035 * | 0.049 * |
| Total clinical staff/Total beds | --NA-- | 0.016 * | 0.042 * |
| COVID-19 occupancy (%) | --NA-- | --NA-- | 0.922 |
| Staff absence (%) | --NA-- | --NA-- | <0.001 *** |
| Influenza occupancy (%) | --NA-- | --NA-- | 0.053 + |
| Random effect: day of week | 0.338 | 0.316 | 0.277 |
| Random effect: patient ID | <0.001 *** | <0.001 *** | <0.001 *** |
| Random effect: Trust code | --NA-- | <0.001 *** | <0.001 *** |
| Spline: date | <0.001 *** | <0.001 *** | <0.001 *** |

Supplementary Table 2. Corresponding p-values for estimated effects for each variable and model. ***, **, *, + refer to p-values below 0.001, 0.01, 0.05 and 0.1 respectively.

| **Model** | **Degrees of Freedom** | **AIC** | **BIC** | **HQIC** |
| --- | --- | --- | --- | --- |
| individual | 19.421 | 94140.713 | **94330.864** | 94197.708 |
| trust | 118.371 | 93577.802 | 94736.767 | 93925.180 |
| pressures | 120.947 | **93559.847** | 94744.038 | **93914.787** |

Supplementary Table 3. Comparison of model degrees of freedom and goodness of fit across the different structures. The model with the lowest AIC fits the data more appropriately accounting for additional parameters in the model. The addition of a trust level random effect increases the BIC of the “trust” model, over the “individual” model. Due to the large number of trusts in the trust level random effect it adds many degrees of freedon, this is penalised most for information criteria that take the sample size into account.


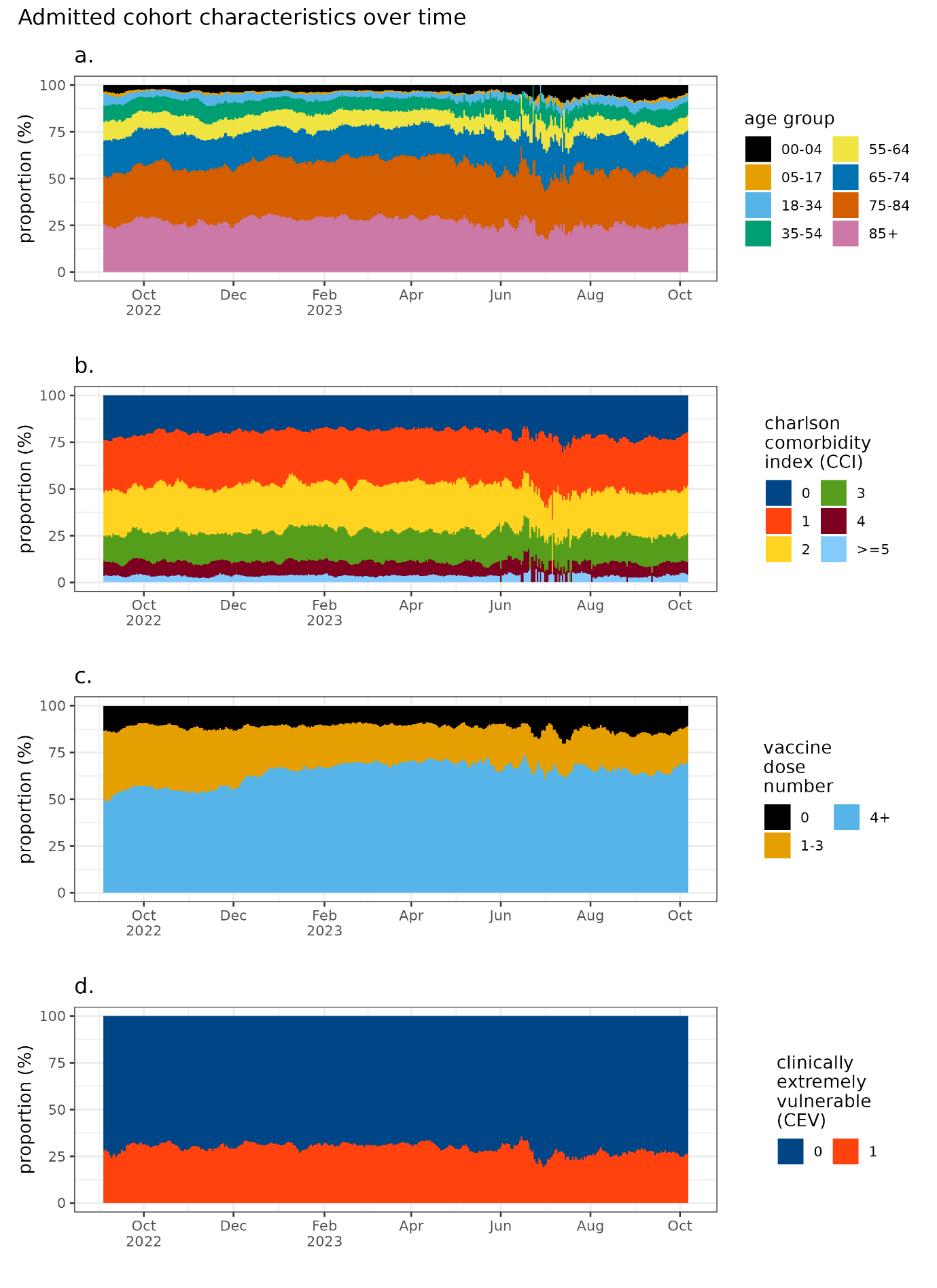


Supplementary Figure 4. The time varying proportion of (a.) age group (b.) Charlson comorbidity index (CCI) (c.) vaccine dose number and (d.) clinically extremely vulnerable (CEV) over the study period. The daily proportions are highly stochastic and have therefore been 7-day centre averaged for ease of interpretation. These plots show the case mix of COVID-19 admissions and how they change over time. There is high variation in the trend between June and August 2023 due to the low counts of admissions.


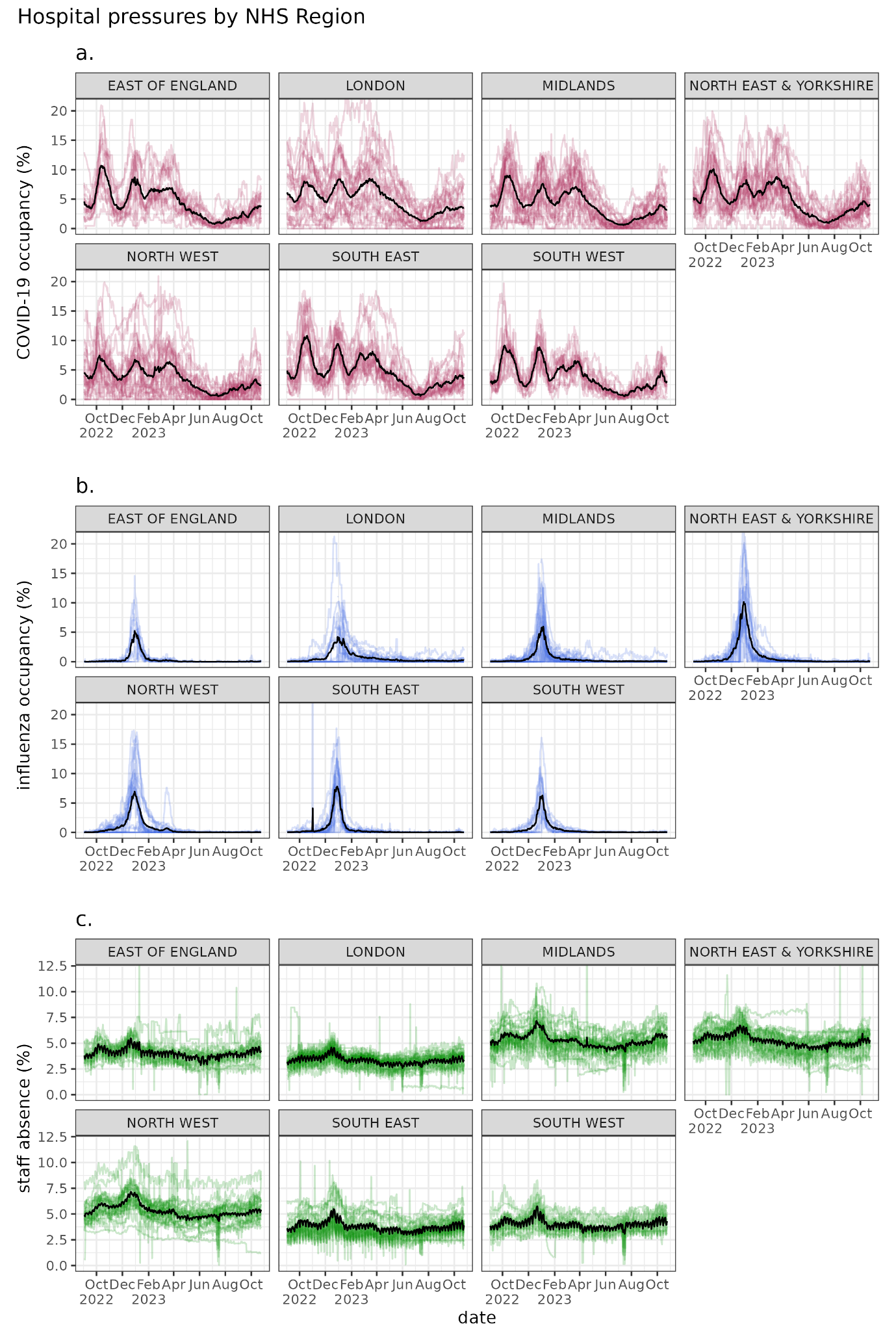


*Supplementary Figure 5. Regional breakdown for the (a.) COVID-19 occupancy (b.) influenza occupancy and (c.) staff absence. There are different levels across regions for the pressures due to differences in local practices and the hierarchical reporting structure of the NHS. The mean value of the pressure regionally is shown with a black line – this is the value imputed for missing values to capture the trend over time and regional practices.*


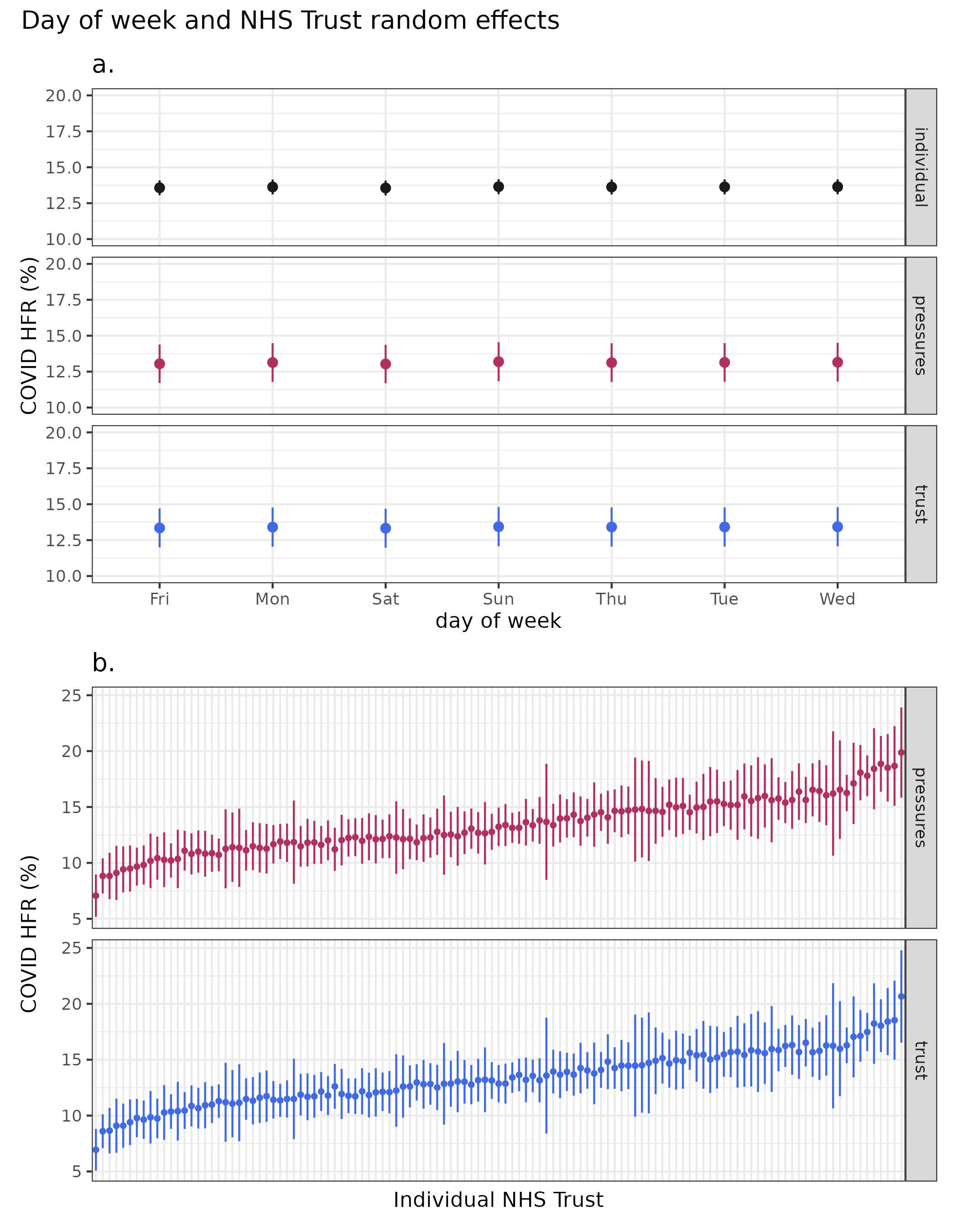


Supplementary Figure 6. The marginal effect of modelled random effects by (a.) day of week and (b.) specific Trusts, ordered by marginal effect. There is no variation in the HFR with the day of week effect across models. The marginal effect of a specific Trust shows a range in estimated average HFRs effects by Trust, indicating different expected outcomes in different hospital locations.
